# Confronting Uncertainty in Wildlife Management: Performance of Grizzly Bear Management

**DOI:** 10.1371/journal.pone.0078041

**Published:** 2013-11-06

**Authors:** Kyle A. Artelle, Sean C. Anderson, Andrew B. Cooper, Paul C. Paquet, John D. Reynolds, Chris T. Darimont

**Affiliations:** 1 Earth to Ocean Research Group, Department of Biological Sciences, Simon Fraser University, Burnaby, British Columbia, Canada; 2 Raincoast Conservation Foundation, Sidney, British Columbia, Canada; 3 School of Resource and Environmental Management, Simon Fraser University, Burnaby, British Columbia, Canada; 4 Department of Geography, University of Victoria, Victoria, British Columbia, Canada; University of Sydney, Australia

## Abstract

Scientific management of wildlife requires confronting the complexities of natural and social systems. Uncertainty poses a central problem. Whereas the importance of considering uncertainty has been widely discussed, studies of the effects of unaddressed uncertainty on real management systems have been rare. We examined the effects of outcome uncertainty and components of biological uncertainty on hunt management performance, illustrated with grizzly bears (*Ursus arctos horribilis*) in British Columbia, Canada. We found that both forms of uncertainty can have serious impacts on management performance. Outcome uncertainty alone – discrepancy between expected and realized mortality levels – led to excess mortality in 19% of cases (population-years) examined. Accounting for uncertainty around estimated biological parameters (*i.e.*, biological uncertainty) revealed that excess mortality might have occurred in up to 70% of cases. We offer a general method for identifying targets for exploited species that incorporates uncertainty and maintains the probability of exceeding mortality limits below specified thresholds. Setting targets in our focal system using this method at thresholds of 25% and 5% probability of overmortality would require average target mortality reductions of 47% and 81%, respectively. Application of our transparent and generalizable framework to this or other systems could improve management performance in the presence of uncertainty.

## Introduction

Confronting uncertainty poses a central problem in the management of wildlife. Decisions made without proper consideration of uncertainty can have undesirable consequences, and have been implicated, for example, in widespread overfishing [1]. Although often poorly accounted for or ignored, uncertainty exists about the “true” value of estimated biological parameters [2], [3], [4], [5]. Parameter uncertainty propagates to uncertainty in important management estimates, including the magnitude of mortality a population can withstand without experiencing long-term declines or other deleterious effects (hereafter “mortality limit uncertainty”) [6], [7]. Management performance can also be compromised by outcome uncertainty, defined as the difference between targeted and realized (*i.e.*, known after the period of exploitation) mortality levels [8]. Remarkably, however, scholarly and independent retrospective examination of wildlife or fisheries management performance – in the presence of uncertainty, or, in general – is rarely conducted (but see [8], [9], [10]).

Several methods can account for and incorporate uncertainty into decision-making, estimating *a priori* the probability that specific scenarios will lead to over-exploitation [1], [2]. Key to implementing these approaches is distinguishing between targets (mortality levels management aims to achieve) and limits (mortality levels management should never exceed). Given that there is always some chance of exceeding a target, management should avoid setting targets as high as limits, or conflating the two [6], [7].

Grizzly bears (*Ursus arctos horribilis*) provide an ideal model species for assessing uncertainty in the management of wildlife. Management of most populations occurs with limited demographic information [11], [12], [13]. Moreover, grizzly bears have life-history characteristics – including long lifespans, low reproductive rates, delayed reproductive maturity, and slow population growth rates [11]– that cause high vulnerability to population declines in many other taxa [14]. Finally, as with many vertebrate taxa [15], mortality is primarily human-caused [11], [16], [17]. As such, management decisions can have considerable influence on population viability [13], [18].

**Figure 1:**
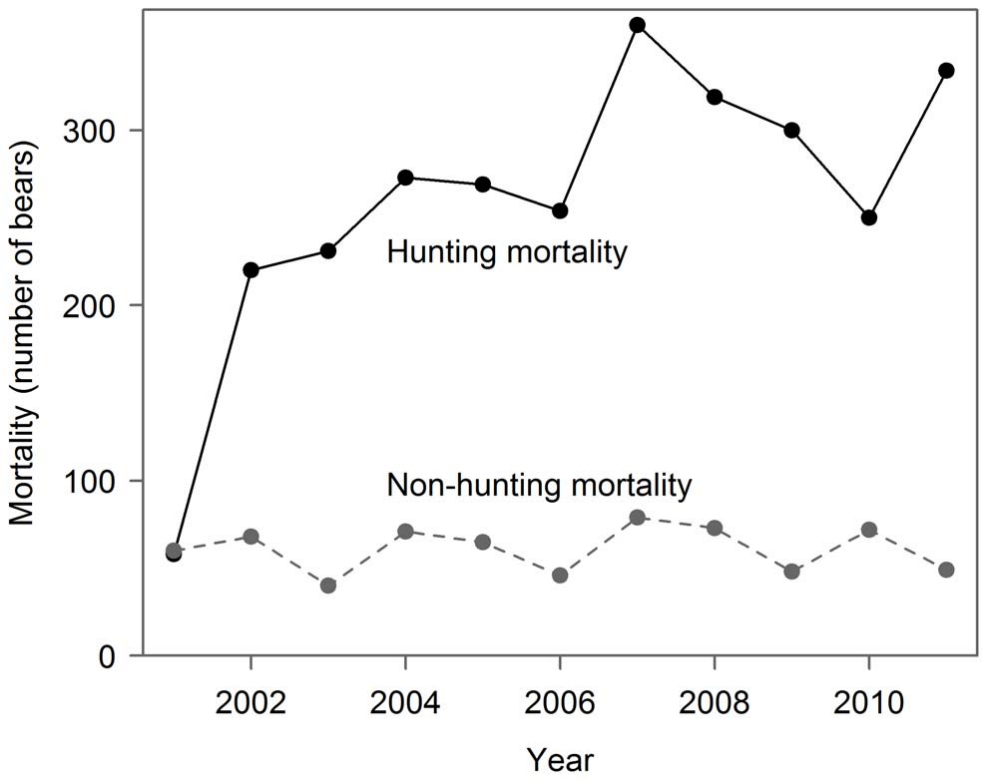
Total grizzly bear (*U. arctos horribilis*) mortality from hunting (solid-black line) and non-hunting sources (dashed line) in British Columbia, Canada, from 2001–2011. A province-wide moratorium on the trophy hunt during one of two hunting seasons caused lower hunting mortality in 2001.

Management of grizzly bear mortality in British Columbia (BC) provides a particularly useful case study for examining effects of uncertainty on management performance. Most populations are managed for sustained yield whereby, in theory, a maximum number of bears (“mortality limit”) can be killed each year by humans, mostly by hunting (Legends Figure 1), without causing population declines [19], [20], [21]. However, uncertainty in mortality limits is only partially addressed by managers in BC; biological parameters and calculated mortality limits are treated as point estimates, with uncertainty adjustments dictated by professional judgement [22], not probabilistic assessments. As such, “true” mortality limits might be lower than suggested [12], [13]. Furthermore, outcome uncertainty is not incorporated; mortality limits are used as mortality targets [20], [23] thereby conflating targets with limits.

Debate about large carnivore management is often contentious and the situation with BC grizzly bears is no exception. Independent scientists have recommended more conservative management [13], [24]. Grizzly bears have been extirpated from a large portion of the province, and, citing sustainability concerns, the European Union has banned the import of BC grizzly bear parts since 2002 [24], [25]. Despite concerns, and concurrent with an increasing number of populations gaining threatened status, hunting mortality increased across the province from 2001–2011 (Legends Figure 1; [26], unpublished data).

We use grizzly bear management in BC from 2001–2011 to explore the potential effects of unaddressed uncertainty on management performance (in our case, the ability to maintain mortality below acceptable limits) and to illustrate general methods for confronting uncertainty in management. Others have highlighted the need to quantitatively address various aspects of uncertainty in management [13], [18], [24]; we add empirical insight by retrospectively assessing historical management. Specifically, we assessed outcome uncertainty by comparing known human-caused mortality with targeted levels. We then used simulation modeling to estimate the biological uncertainty around mortality limit point estimates based on parameter uncertainty and assessed how mortality limit uncertainty might affect overmortality probabilities. Finally, we incorporated outcome and mortality limit uncertainty into a generalizable and transparent method for identifying mortality targets that maintain the probability of overmortality below pre-determined thresholds. We discuss how this general approach might help inform population management of other exploited species.

## Methods

We conducted our analyses at the Grizzly Bear Population Unit (hereafter “population unit”) spatial scale, thought to reflect ecologically and demographically relevant sub-populations [21]. We divided our study period into the same multi-year allocation periods (2001–2003, 2004–2006, and 2007–2011) used by the British Columbia Ministry of Environment (hereafter “government”; [21]). We calculated known mortality for each population unit and each allocation period using a government database (“Compulsory Inspection Database”) of all known human-caused mortality including licensed hunting, animal control kills, road and rail accidents, and known poaching [21]. Additionally, we followed government procedures for calculating mortality limits (in units of bears per allocation period) based on estimates of population size, annual allowable mortality (AAM; proportion of a population that can theoretically be removed without causing population declines), and unreported mortality (from rates observed in one population unit and extrapolated to other population units based on four variables thought to correlate with unreported mortality; See Appendix S1). In our outcome uncertainty analyses we applied the government’s “uncertainty correction factors” to population estimates, whereas in subsequent analyses we used an empirical and probabilistic approach to address uncertainty. In most population units, the correction factors used by BC managers are deterministic values, based on expert judgement, that are inversely proportional to estimated population sizes (Appendix S1, [23]). Our analyses followed the government practice of calculating mortality limits for the entire population (Eq 1) and for females separately (Eq 2) to account for the sensitivity of populations to female mortality [19], [21], [27]. We also calculated female mortality as a percentage of total mortality. The government subtracts predicted non-hunt mortality (*e.g.* road kill, animal control kills, and illegal hunting) estimates from mortality limits and allocates the remaining mortality to hunting. We note, however, that by allocating mortality right up to mortality limits, BC managers treat limits as targets, conflating the two; we hereafter refer to true targeted mortality levels (whether or not they are conflated with mortality limits by managers) as “targets” and true, biologically-determined mortality limits as “limits”. Details on mortality limit calculations, and on how they differed among periods, are provided in Appendices S1 and S2, respectively.
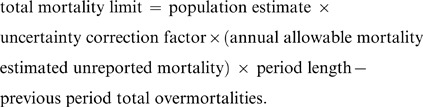
(1)

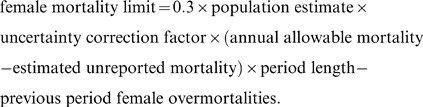
(2)


### Outcome Uncertainty and Mortality Patterns

We assessed outcome uncertainty across population units and across study periods by calculating the difference between known mortality (from the Compulsory Inspection Database) and targeted mortality:
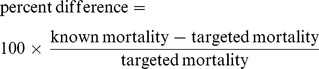
(3)


We further explored patterns of mortality types associated with overmortality events.

We characterized outcome uncertainty as a function of targeted mortality. Using maximum likelihood estimation, we fit Michaelis-Menton curves to model known mortality as a function of targeted mortality, for each period, and for total and female mortality:
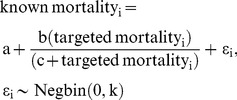
(4)where i represents a population unit-period; **a**, **b**, **c**, are estimated parameters of the curve; 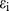 represents residual error; and **k** is the estimated size parameter of a negative binomial error distribution with a mean of 0. We used this error distribution because targets must be positive integer values. We fit the models using *optim* in R 2.14.1 (R Core Team 2012, R Foundation for Statistical Computing) with the Nelder-Mead method and with estimated parameters in log space.

### Mortality Limit Uncertainty and Probability of Overmortality

**Figure 2:**
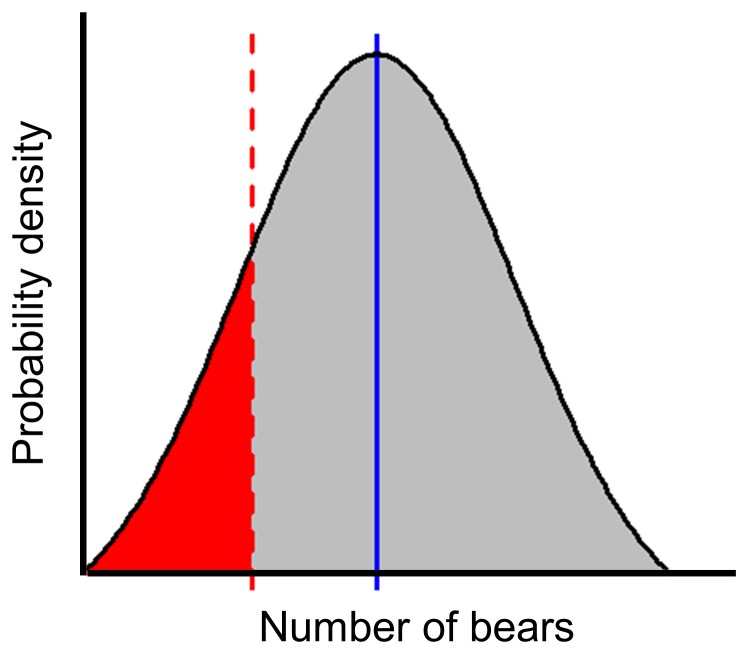
Illustration of method for estimating the probability of overmortality in an individual Grizzly Bear (*U. arctos horribilis*) Population Unit (“population unit”) and period. Blue vertical line represents the mortality limit point estimate used by government. Entire distribution (in this example a hypothetical normal distribution used for simplicity) represents the distribution of mortality limit uncertainty, or the distribution of simulated mortality limits. Red dashed line represents the known mortality for the same population unit-period. Red portion of the distribution represents the proportion of simulated mortality limits that fell below known mortality levels in the population unit-period. The percent area of the overall distribution occupied by the red portion provides a proxy for the probability that overmortality occurred. See also Video S1.

Whereas current management procedure (above) treats mortality limits as point estimate, we propagated biological parameter uncertainty to estimate cumulative uncertainty around mortality limits using simulation modeling [28], [29] and assessed how this uncertainty might affect the probability of overmortality. We focused on three key parameters currently treated as point estimates by managers. Because empirically derived uncertainty estimates are lacking for most BC populations, we derived parameter uncertainty estimates from a literature review (Appendix S3). For each parameter, we took random draws from a continuous uniform distribution centered on existing point estimates. The distributions were bounded by: population estimates: +/−40% of point estimate; AAM: +/−2% of population estimate (because AAM is a percentage of population estimate); and unreported mortality: from 50% (*i.e.* half) to 200% (*i.e*., double) of the point estimate (Appendix S3). We calculated simulated female and total mortality limits by substituting randomly drawn parameter values into Eq 1 and Eq 2. We did not incorporate the government’s estimated uncertainty correction factors in these calculations. We repeated these simulations 1000 times in each population unit and period to construct a distribution of realistic mortality limits (the simulated breadth of mortality limit uncertainty). We used the percentage of simulations in which simulated mortality limits fell below known mortalities as a proxy for overmortality probability (Figure 2, Video S1).

### Identifying Targets that Incorporate Outcome and Mortality Limit Uncertainty

We used derived distributions of outcome and mortality limit uncertainty to calculate targets for each population unit that maintained the probability of overmortality below 5% (low risk-tolerant, conservation-prioritizing scenario) or 25% (higher risk-tolerant, exploitation-prioritizing scenario), using data from 2007–2011. For a given target, we used a “plug-in” approach [30] to estimate outcome uncertainty. This approach estimates outcome uncertainty from the stochastic component (the negative binomial error) of Eq 4, assuming that the deterministic component (the Michaelis-Menten curve) was fixed at the maximum likelihood estimate. For each population unit, we calculated the intersection of the resultant outcome uncertainty and mortality limit distributions for all possible target values, keeping mortality limit distributions fixed, to find the highest target for which the resultant outcome uncertainty distribution intersected with less than the maximum area (the given thresholds, 5% or 25%) of the mortality limit distribution (Video S2). We performed all analyses with R 2.14.1 (R Core Team 2012, R Foundation for Statistical Computing).

## Results

### Outcome Uncertainty and Mortality Patterns

**Figure 3:**
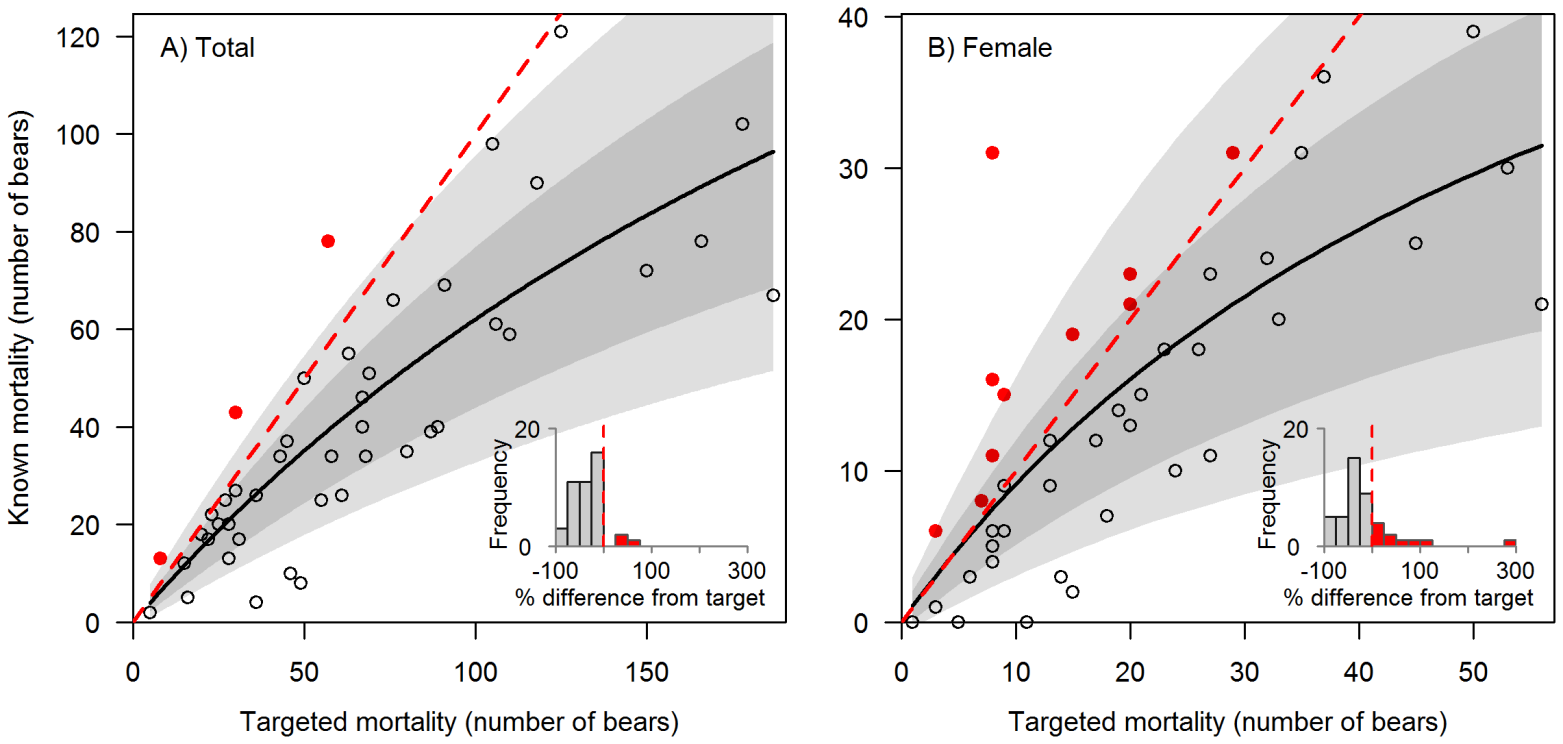
Outcome uncertainty for A) total and B) female mortality in Grizzly Bear (*U. arctos horribilis*) Population Units (“population units”) in British Columbia, Canada, 2007–2011 (see SI for additional periods). Black curve is a Michaelis-Menten curve fitted by maximum likelihood, assuming a negative binomial error distribution. Red dashed line indicates a 1∶1 relationship; solid red dots above this line signal population unit-level overmortality events. Dark and light grey-shaded regions encompass the 50% and 80% prediction intervals, respectively (smoothed for visual purposes). Inset histograms show the distribution of GBPU-level percent difference between known mortalities and mortality targets (conflated with limits under mortality management policy); red bars to the right of red dashed lines indicate overmortality events.

**Figure 4:**
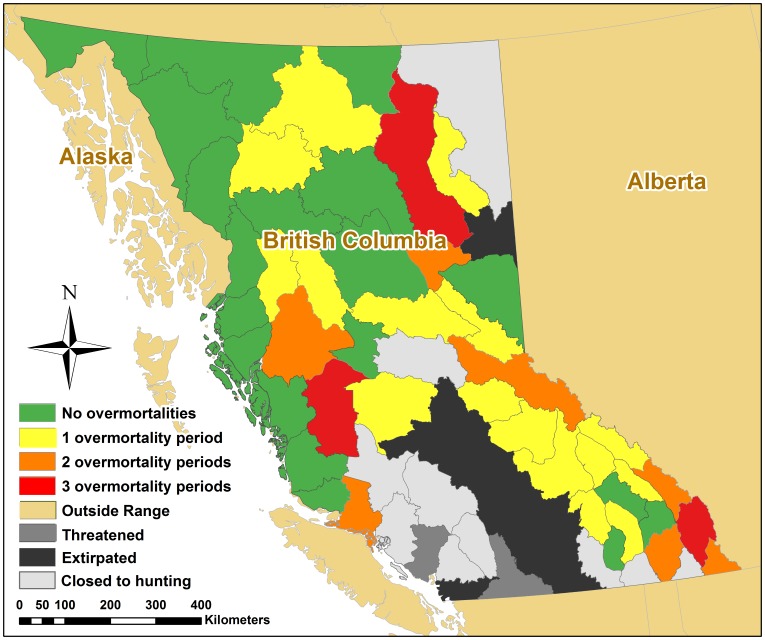
Number of allocation periods (2001–2003, 2004–2006, or 2007–2011) in which female or total overmortality occurred in Grizzly Bear (*U. arctos horribilis*) Population Units (“population units”) of British Columbia, Canada. Shown are 2009 population unit boundaries. Hunting is not allowed in areas denoted as “threatened”, “extirpated”, or “closed to hunting”. One additional population unit (Blackwater-West Chilcotin) has been reclassified as threatened as of 2012.

Outcome uncertainty varied across population units and periods, with discrepancies between targeted and known mortality being more pronounced for female mortalities than total mortalities (Figure 3, S1, and S2). Because government procedures conflated targets with limits, cases in which targets were exceeded also constituted overmortalities. While mortality fell mostly below targets, overmortalities occurred in at least one period in 26 of the approximately 50 population units open for hunting, and most frequently in southern and eastern BC (Figure 4). Overmortalities (18 total cases and 33 female cases from 2001–2011) occurred more frequently in population units with smaller targets (Figure 3, S1, S2, and S3). In seven population units, overmortality events occurred in two periods, whereas in three population units they occurred in all three periods (Figure 4). Overmortality events ranged from one to 24 bears. Finally, targets were also frequently approached but not exceeded (Figure S3).

The most common factor associated with total overmortalities was unpredicted non-hunting mortality. However, most of the total overmortalities from 2001–2011 (17 of 18, or 94%) could have been avoided with reduced hunting mortality (Figure S3). The most common factor associated with female overmortalities was hunting mortality. Most female overmortalities (25 of 33, or 76%) could have been avoided with reduced hunting mortality (Figure S3).

**Figure 5:**
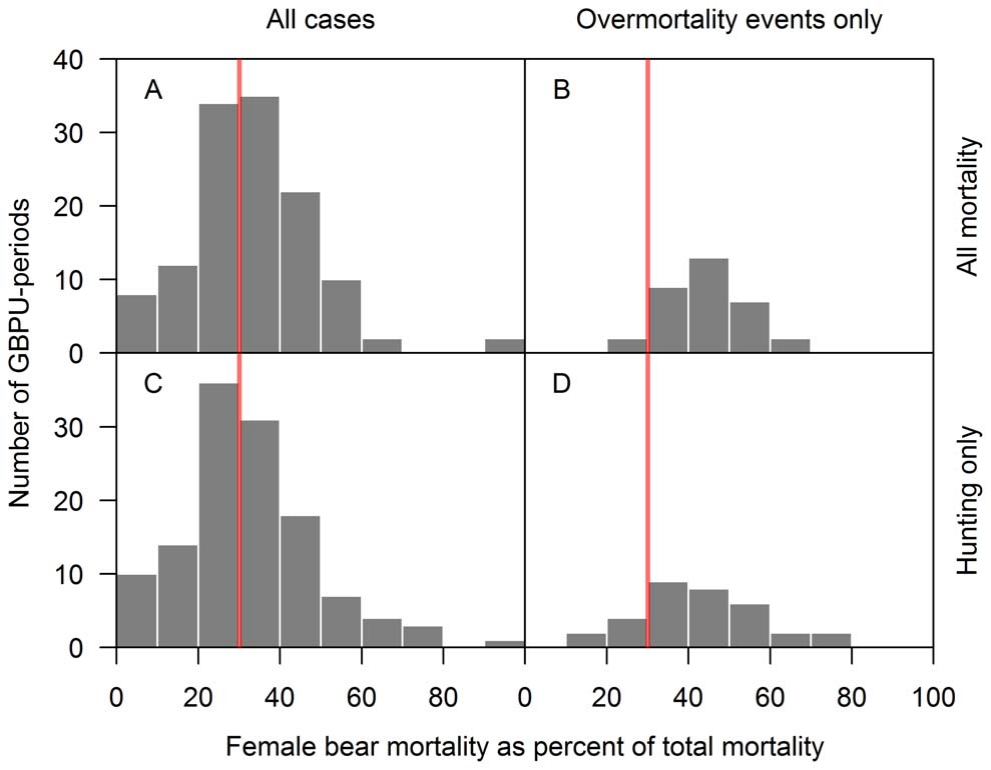
Female mortality as percent of total mortality across Grizzly Bear (*U. arctos horriblis)* Population Units (“population units”) in British Columbia, Canada, and allocation periods (2001–2003, 2004–2006, and 2007–2011). (A) female mortality as percent of all mortality, (B) female mortality as percent of all mortality in female overmortality events, (C) female hunting mortality as percent of all hunting mortality, and (D) female hunting mortality as a percent of all hunting mortality in female overmortality events. Vertical red lines indicate 30%, the threshold below which female mortality must remain for total mortality limits to be theoretically sustainable according to the BC government’s mortality management procedure.

The female component exceeded 30% of *total* mortality (from hunting and non-hunting sources combined) in 55% of all cases and in 94% of all female overmortality events (Figure 5 A and B, respectively). The female component exceeded 30% of *total hunting* mortality in 50% of all cases and in 82% of all female overmortality cases (Figure 5 C and D, respectively).

### Mortality Limit Uncertainty and Probability of Overmortality

**Figure 6:**
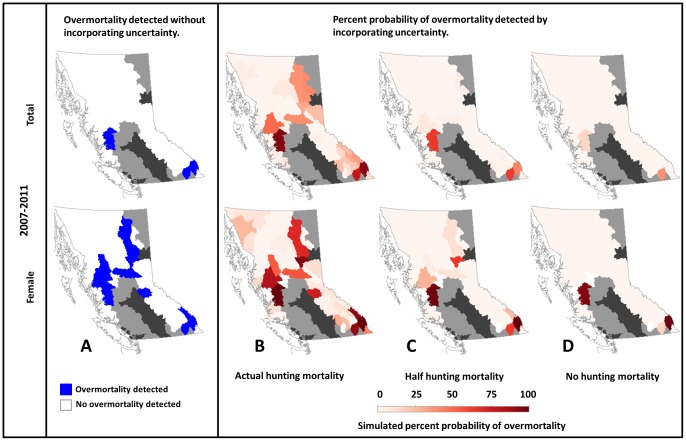
Total and female overmortalities of Grizzly Bear (*U. arctos horribilis*) Population Units (“population units”) of British Columbia, Canada, from 2007–2011 (see SI for additional periods). A) Overmortalities detected given known hunting mortality levels and without consideration of mortality limit uncertainty. Blue indicates population units with detected overmortality whereas white indicates population units without. B–D) Simulated probability of total or female overmortality, incorporating uncertainty around mortality limits. Panel B shows simulated probability of overmortality given known mortality rates; panels C and D show what the probability of overmortality would be had hunting mortality been reduced by 50% or 100%, respectively, assuming other sources of mortality remained unchanged. Increasingly dark red indicates an increasing probability of overmortality in a given period. Grizzly bears have been extirpated from dark-grey areas. Light-grey areas indicate population units in which populations are either threatened or were closed to hunting during the study period.

Accounting for components of biological uncertainty revealed that overmortalities might have occurred in 90 of 127 (71%) examined female cases and 89 of 127 (70%) examined total cases. This comprised an additional 45% of female cases and 56% of total cases relative to overmortality assessments that did not consider uncertainty (Figures 6 A and B, S4 A and B, and S5 A and B). Even in the face of uncertainty, reducing hunting by half would have reduced the probability of overmortality by an average of 85% for total and 75% for female overmortality cases (Figures 6C, S4C, and S5C), whereas completely eliminating hunting would have reduced the probability of overmortality by an average of 96% for total and 89% for female overmortality cases (Figures 6D, S4D, and S5D).

### Identifying Targets that Incorporate Outcome Uncertainty and Mortality Limit Uncertainty

**Figure 7:**
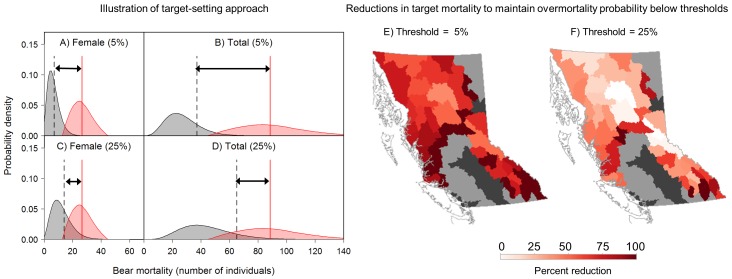
Illustration of our method for setting female (A and C) and total (B and D) mortality targets, with maximum probability of overmortality of 5% (A and B) or 25% (C and D) by integrating outcome uncertainty (grey distribution) and mortality limit uncertainty (red distribution), using the Stewart Grizzly Bear (*U. arctos horribilis*) Population Unit as an example. Targets (dashed black lines) from this approach are set so that the resulting outcome uncertainty distribution (grey distribution) overlaps with a maximum of 5% or 25% of the mortality limit uncertainty distribution (red distribution). Red vertical lines represent mortality limits (conflated with targets in previous periods under mortality management policy) set by the government in 2007–2011. Magnitudes of recommended target reductions are shown by black double-headed arrows. E-F) Reduction in mortality targets, relative to 2007–2011 targets (conflated with limits under mortality management policy), required to maintain the probability of both female and total overmortality below E) 5% or F) 25%. Increasingly dark red indicates increasing target reductions identified.

To maintain the probability of overmortality below a 5% threshold, mortality targets would need to be reduced by an average of 81% across all population units relative to 2007–2011 targets, and by 100% in 15 (Figure 7 A, B and E). For the exploitation-prioritizing 25% threshold, mortality targets would still need to be reduced by an average of 47% across all population units, and by 100% in four population units (Figure 7 C, D, and F).

## Discussion

Our analysis illustrates the importance of assessing management performance and uncertainty. Specifically, we found that unaddressed uncertainty could compromise management performance by leading to excessive mortalities in hunted species. We found that grizzly bear overmortalities in British Columbia, Canada, were spatially widespread, occurred repeatedly, and were more frequent in females. Considering biological uncertainty around mortality limits revealed that many additional populations might have experienced overmortalities. A target-setting framework that incorporates outcome and mortality limit uncertainty shows that considerable reductions in targeted mortality would be required to improve management performance.

### Considerations

We used grizzly bears to illustrate general issues applicable to many other taxa, rather than prescribing specific management actions for this particular species. Moreover, mortality limit simulations used uniform distributions with ranges considerably narrower than the full extent suggested in the literature (see Appendix S3 for full ranges). We had insufficient data to determine clearly which particular distribution best approximated such parameters; however, the use of such limited ranges of uncertainty suggests our estimates of overmortality risks and target reductions were underestimated even if the true error structure followed a different distribution (*e.g.* normal or log-normal). Importantly, estimated probabilities of overmortality and reductions in targeted mortalities would change if empirically derived and area-specific ranges and distributions of uncertainty were known for each population unit. Similarly, given that the outcome uncertainty was estimated from management performance over a short time, our derived distributions likely underestimated the true range of uncertainty. Additionally, the relationship between targeted and known mortality changes through time (as might be expected given the fluidity of political, social, and ecological contexts, for example), which potentially affects the ability to predict the future using historical data. However, by frequently and iteratively re-evaluating management performance, managers adopting this approach could detect such changes and respond by updating outcome uncertainty distributions. Finally, our analyses did not address assumptions used by management in setting specific parameter point estimates for each area, or in adjusting estimates among periods, which could have affected our ability to detect overmortalities (Appendix S2). Given these considerations, our results could provide minimal requirements for improving performance in this particular system; we recommend that management systems adapting this approach obtain geographically-explicit data, and characterize and incorporate uncertainty. We also recommend that management be re-evaluated, updated, and refined iteratively to account for possible changes in dynamics in targeted species and hunter behaviour.

### Additional Sources of Uncertainty

Our analyses addressed only a subset of uncertainty in the management of wildlife. For example, there is additional uncertainty about the appropriateness of models used in setting limits (“model selection error”; [1]); genetic, phenotypic, or social effects of exploitation on hunted populations (e.g. [31], [32], [33]); time required for population recovery [14]; effects of declining food availability [34]; and the cumulative effect of other anthropogenic disturbances such as logging, mineral extraction, roads, and development [12], [24], [35]. Despite examining only a subset of uncertainty, our work empirically illustrates potential effects on management performance, and suggests methods management agencies could consider.

### Management Performance and Outcome Uncertainty

Multiple processes may contribute to outcome uncertainty. For instance, in the case of grizzly management, hunting mortality, especially in females, was often higher than targeted. Guidelines that encourage hunters to avoid females seem inadequate given that female mortality consistently exceeded the 30% threshold dictated by government procedures [21], [23], [27]. Similar barriers to limiting female mortality might also apply to other wildlife species in which sexes are not particularly dimorphic, with similar concerns about population dynamics (e.g. caribou *Rangifer tarandus*, [36]). Additionally, although most total and female overmortality events could have been prevented through hunting reductions, mortality sources beyond management control might also contribute to outcome uncertainty. In our analysis road kill, animal control kills, and illegal hunting were important, highlighting that measures beyond hunt reductions are likely required to safeguard populations. Importantly, not explicitly incorporating outcome uncertainty into procedures for management of wildlife could result not only in sporadic and isolated, but also chronic and repeated, overmortality events, as highlighted in our study period in which overmortalities occurred repeatedly in many areas.

### Mortality Limit Uncertainty

In addition to outcome uncertainty, uncertainty not explicitly accounted for in estimating biological parameters, such as mortality limits, can also lead to excessive mortality. For example, by accounting for mortality limit uncertainty, our simulations revealed that overmortality events might have occurred in many cases in which mortality did not exceed government-determined mortality limits. We found that the probability of overmortality would have decreased considerably had hunting been reduced or eliminated, as expected for any system in which hunting constitutes most mortality. This result provides management a direct and easily controlled route to reducing the probability of over-exploitation.

### Identifying Targets that Incorporate Uncertainty

Our framework for transparently incorporating uncertainty identified targets that reduce the probability of over-exploitation. This approach is a considerable improvement from the deterministic and ad hoc “uncertainty correction factors” used in previous management. In our approach, uncertainty is incorporated in a repeatable, quantitative and transparent fashion, and can readily include new data as they become available. Of particular relevance to managers, the public, and decision-makers is how mortality management might change if this approach were implemented. Our simulations revealed that careful management would require considerable target reductions, consistent with the conservative ‘bet-hedging’ recommended for cautious management [2], [12]. Importantly, given that female mortality seems difficult to control independently of total mortality, a given population unit’s total target mortality would need to be reduced sufficiently to maintain total and female overmortality probabilities below thresholds. Recommended targets changed considerably depending on the threshold used, highlighting the importance of careful consideration and engagement of stakeholders when setting targets. Although the acceptable probabilities of overmortality used in our approach (5% or 25%) were arbitrary, they might represent thresholds for a low risk-tolerant, conservation prioritizing scenario and a higher risk-tolerant, exploitation-prioritizing scenario, respectively. Notably, hunting reductions would be required even in the exploitation-prioritizing scenario.

### Identifying Targets in Other Scenarios

Our case study illustrated an approach for reducing the risk of *over*mortality of species managed for long-term population viability. This approach could also be used for reducing the risk of *under*mortality of species managed for population reduction or elimination, such as in the control or eradication of invasive species (*e.g.* control of invasive lionfish through exploitation [37]). In such cases targets would be set sufficiently *high* to ensure they do not fall below levels needed to obtain population reductions required. This approach provides the first steps to a full decision analysis framework, a quantitative approach for weighing various management options that might be appropriate in future management deliberations [2], [29].

### Importance of Incorporating Best-practices from Other Disciplines

This study illustrates the merit of incorporating approaches from other disciplines and taxa into wildlife management. Whereas BC grizzly bear management incorporates data and management techniques from grizzly bear management in other jurisdictions [19], [21], it does not incorporate some promising methods from other disciplines. For example, our approach, which relies on the principle that targets should be set sufficiently low to account for uncertainty (and lower than most of the estimated range of mortality limits; [2], [6], [7]) is used in fisheries but far less commonly in wildlife management, highlighting the need for better integration of best practices across taxa and disciplines.

## Conclusion

Science can provide valuable insight into management issues often mired in heated debate. Management often occurs within contentious social environments, with interest groups advocating strongly for different scenarios, informed by varying ethical perspectives and philosophies [10], [38], [39], [40], [41]. Science can inform such debate by assessing the ability of management to achieve objectives and by transparently communicating risks associated with various scenarios. We suggest that many management systems might benefit from retrospective and empirical examinations that can inform present and future management. These could be conducted as a part of the management process or, as in this study, by third parties. Results and predictions from such examinations in any system could help to communicate likely outcomes while simultaneously improving future management performance.

## Supporting Information

Figure S1 **Outcome uncertainty for A) total and B) female mortality in Grizzly Bear (** ***U. arctos horribilis*** **) Population Units (“population units”) in British Columbia, Canada, 2001–2003 (see SI for additional periods).** Black curve is a Michaelis-Menten curve fitted by maximum likelihood, assuming a negative binomial error distribution. Red dashed line indicates a 1∶1 relationship; solid red dots above this line signal population unit-level overmortality events. Dark and light grey-shaded regions encompass the 50% and 80% prediction intervals, respectively (smoothed for visual purposes). Inset histograms show the distribution of GBPU-level percent difference between known mortalities and mortality limits (conflated with limits under mortality management policy); red bars to the right of red dashed lines indicate overmortality events.
(TIF)

Figure S2 **Outcome uncertainty for A) total and B) female mortality in Grizzly Bear (** ***U. arctos horribilis*** **) Population Units (“population units”) in British Columbia, Canada, 2004–2006.** Black curve is a Michaelis-Menten curve fitted by maximum likelihood, assuming a negative binomial error distribution. Red dashed line indicates a 1∶1 relationship; solid red dots above this line signal population unit-level overmortality events. Dark and light grey-shaded regions encompass the 50% and 80% prediction intervals, respectively (smoothed for visual purposes). Inset histograms show the distribution of GBPU-level percent difference between known mortalities and mortality targets (conflated with limits under mortality management policy); red bars to the right of red dashed lines indicate overmortality events.
(TIF)

Figure S3 **Mortality targets (conflated with limits under mortality management policy) and known mortalities for each Grizzly Bear (** ***U. arctos horribilis*** **) Population Unit (population unit) in British Columbia, Canada, during A) 2001–2003, B) 2004–2004, and C) 2007–2011 allocation periods.** Green and orange bars represent number of bears killed by non-hunting and hunting sources, respectively. Vertical grey lines denote mortality targets and vertical black lines denote predicted non-hunt mortality for each period. Population unit rows in which known mortality exceeded mortality targets (‘overmortality’) are shown with grey highlighting. Open blue circles denote population units in which hunting mortality alone exceeded the mortality targets for all sources combined; filled blue circles denote areas in which the unpredicted non-hunting mortality (difference between known and predicted non-hunting mortality) exceeded the excess mortality.
(TIF)

Figure S4 **Total and female overmortalities of Grizzly Bear (** ***U. arctos horribilis*** **) Population Units (“population units”) of British Columbia, Canada, from 2001–2003.** A) Overmortalities detected given known hunting mortality levels and without consideration of mortality limit uncertainty. Blue indicates population units with detected overmortality whereas white indicates population units without. B–D) Simulated probability of total or female overmortality, incorporating uncertainty around mortality limits. Panel B shows simulated probability of overmortality given known mortality rates; panels C and D show what the probability of overmortality would be had hunting mortality been reduced by 50% or 100%, respectively, assuming other sources of mortality remained unchanged. Increasingly dark red indicates an increasing probability of overmortality in a given period. Grizzly bears have been extirpated from dark-grey areas. Light-grey areas indicate population units in which populations are either threatened or were closed to hunting during the study period.
(TIF)

Figure S5 **Total and female overmortalities of Grizzly Bear (** ***U. arctos horribilis*** **) Population Units (“population units”) of British Columbia, Canada, from 2004–2006.** A) Overmortalities detected given known hunting mortality levels and without consideration of mortality limit uncertainty. Blue indicates population units with detected overmortality whereas white indicates population units without. B–D) Simulated probability of total or female overmortality, incorporating uncertainty around mortality limits. Panel B shows simulated probability of overmortality given known mortality rates; panels C and D show what the probability of overmortality would be had hunting mortality been reduced by 50% or 100%, respectively, assuming other sources of mortality remained unchanged. Increasingly dark red indicates an increasing probability of overmortality in a given period. Grizzly bears have been extirpated from dark-grey areas. Light-grey areas indicate population units in which populations are either threatened or were closed to hunting during the study period.
(TIF)

Appendix S1 (DOCX)

Appendix S2 (DOCX)

Appendix S3 (DOCX)

Video S1 (WMV)

Video S2 (WMV)
