# Supplementary figures and images for: Confronting Uncertainty in Wildlife Management: Performance of Grizzly Bear Management
Source: PLoS One. 2013 Nov 6;8(11):e78041. doi: 10.1371/journal.pone.0078041 (PMC3819331; doi:10.1371/journal.pone.0078041)

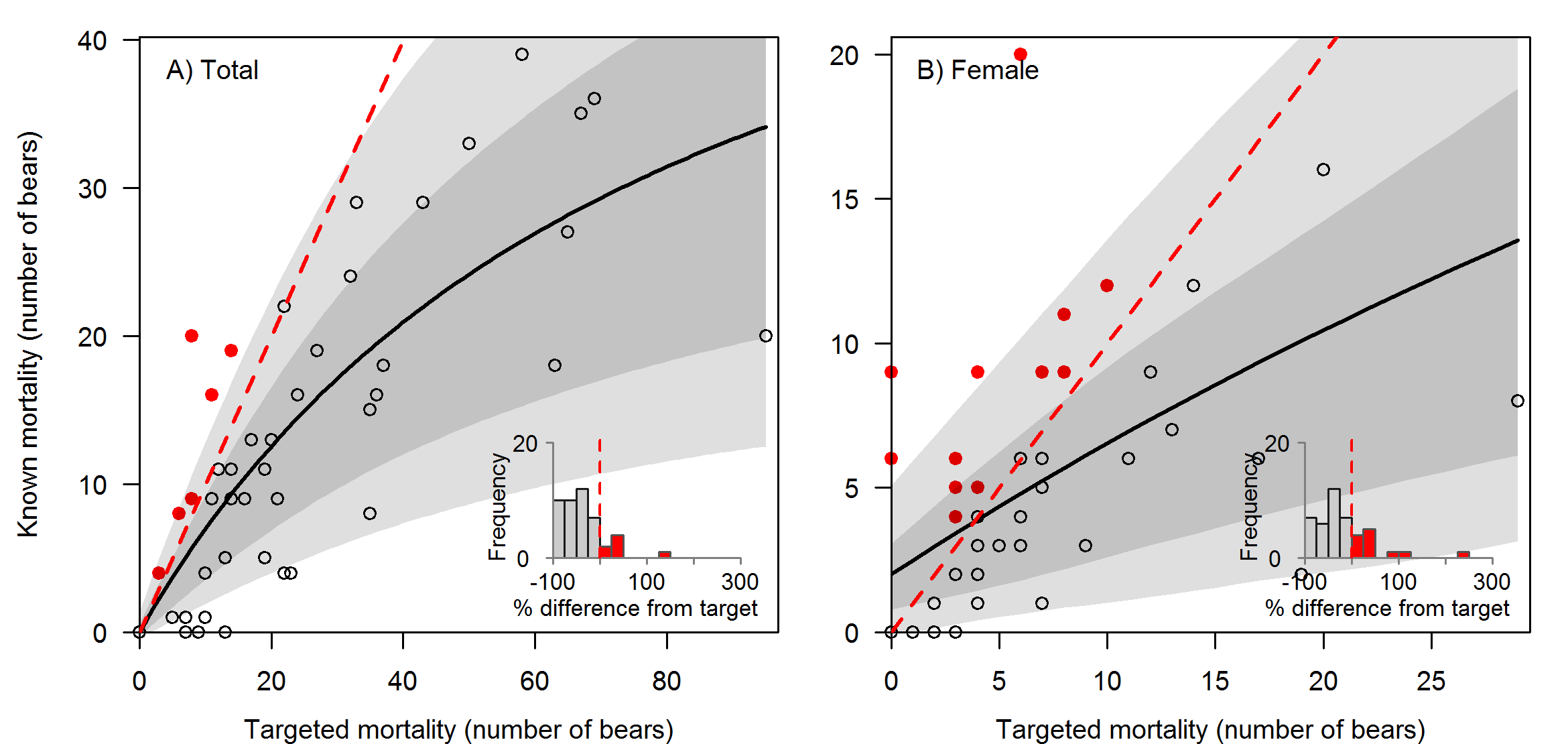

Supplement: Figure S1 — Outcome uncertainty for A) total and B) female mortality in Grizzly Bear ( U. arctos horribilis ) Population Units (“population units”) in British Columbia, Canada, 2001–2003 (see SI for additional periods). Black curve is a Michaelis-Menten curve fitted by maximum likelihood, assuming a negative binomial error distribution. Red dashed line indicates a 1∶1 relationship; solid red dots above this line signal population unit-level overmortality events. Dark and light grey-shaded regions encompass the 50% and 80% prediction intervals, respectively (smoothed for visual purposes). Inset histograms show the distribution of GBPU-level percent difference between known mortalities and mortality limits (conflated with limits under mortality management policy); red bars to the right of red dashed lines indicate overmortality events. (TIF) [file pone.0078041.s001.tif]

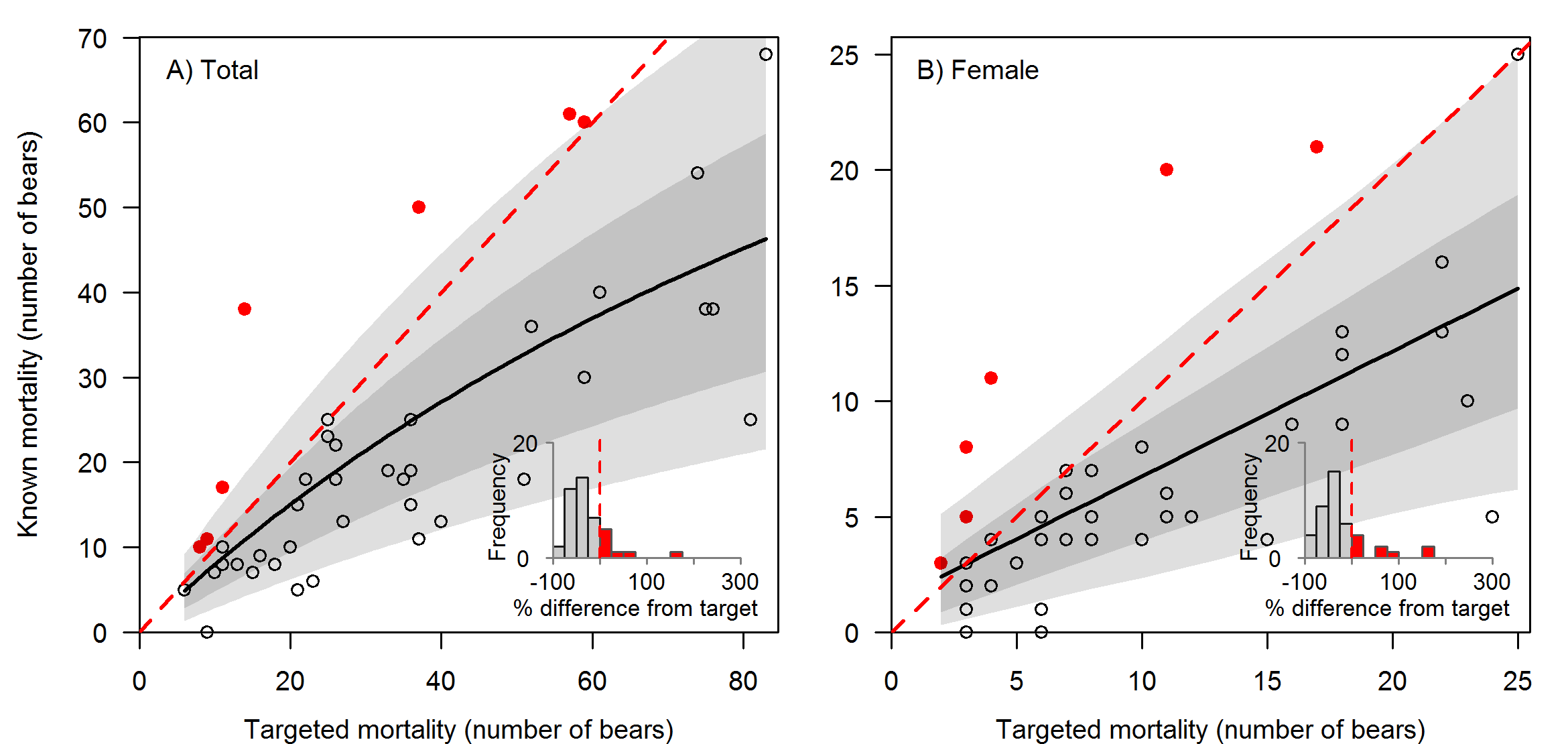

Supplement: Figure S2 — Outcome uncertainty for A) total and B) female mortality in Grizzly Bear ( U. arctos horribilis ) Population Units (“population units”) in British Columbia, Canada, 2004–2006. Black curve is a Michaelis-Menten curve fitted by maximum likelihood, assuming a negative binomial error distribution. Red dashed line indicates a 1∶1 relationship; solid red dots above this line signal population unit-level overmortality events. Dark and light grey-shaded regions encompass the 50% and 80% prediction intervals, respectively (smoothed for visual purposes). Inset histograms show the distribution of GBPU-level percent difference between known mortalities and mortality targets (conflated with limits under mortality management policy); red bars to the right of red dashed lines indicate overmortality events. (TIF) [file pone.0078041.s002.tif]

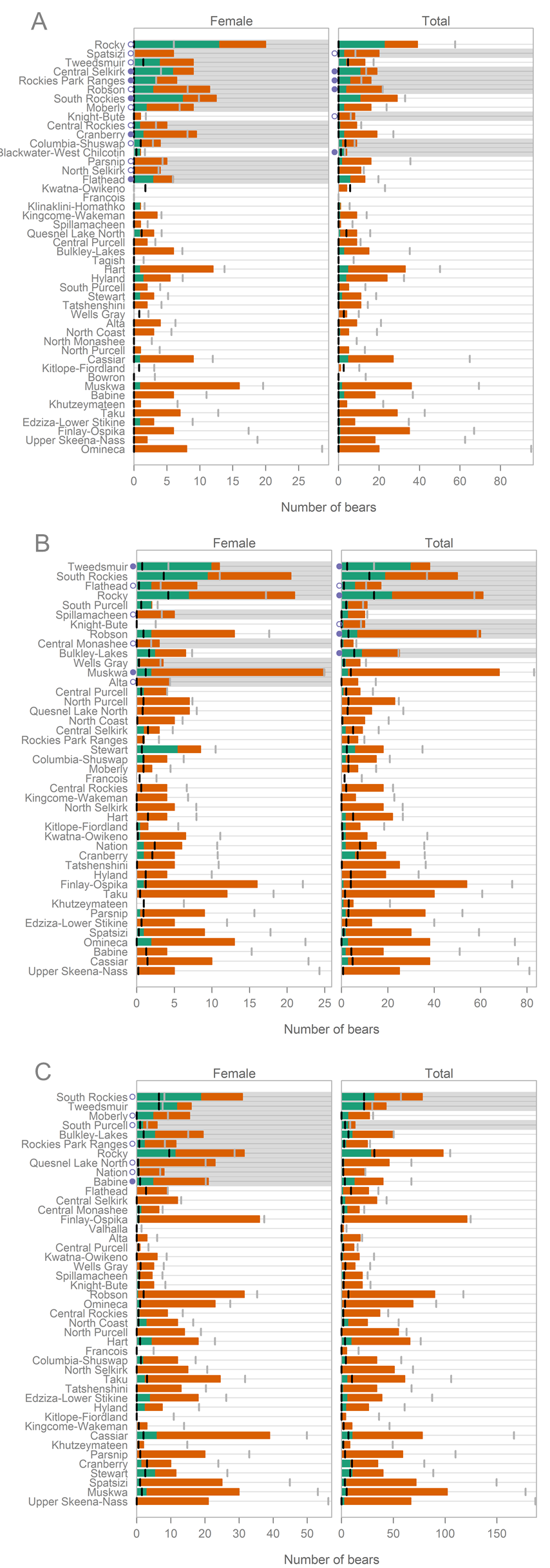

Supplement: Figure S3 — Mortality targets (conflated with limits under mortality management policy) and known mortalities for each Grizzly Bear ( U. arctos horribilis ) Population Unit (population unit) in British Columbia, Canada, during A) 2001–2003, B) 2004–2004, and C) 2007–2011 allocation periods. Green and orange bars represent number of bears killed by non-hunting and hunting sources, respectively. Vertical grey lines denote mortality targets and vertical black lines denote predicted non-hunt mortality for each period. Population unit rows in which known mortality exceeded mortality targets (‘overmortality’) are shown with grey highlighting. Open blue circles denote population units in which hunting mortality alone exceeded the mortality targets for all sources combined; filled blue circles denote areas in which the unpredicted non-hunting mortality (difference between known and predicted non-hunting mortality) exceeded the excess mortality. (TIF) [file pone.0078041.s003.tif]

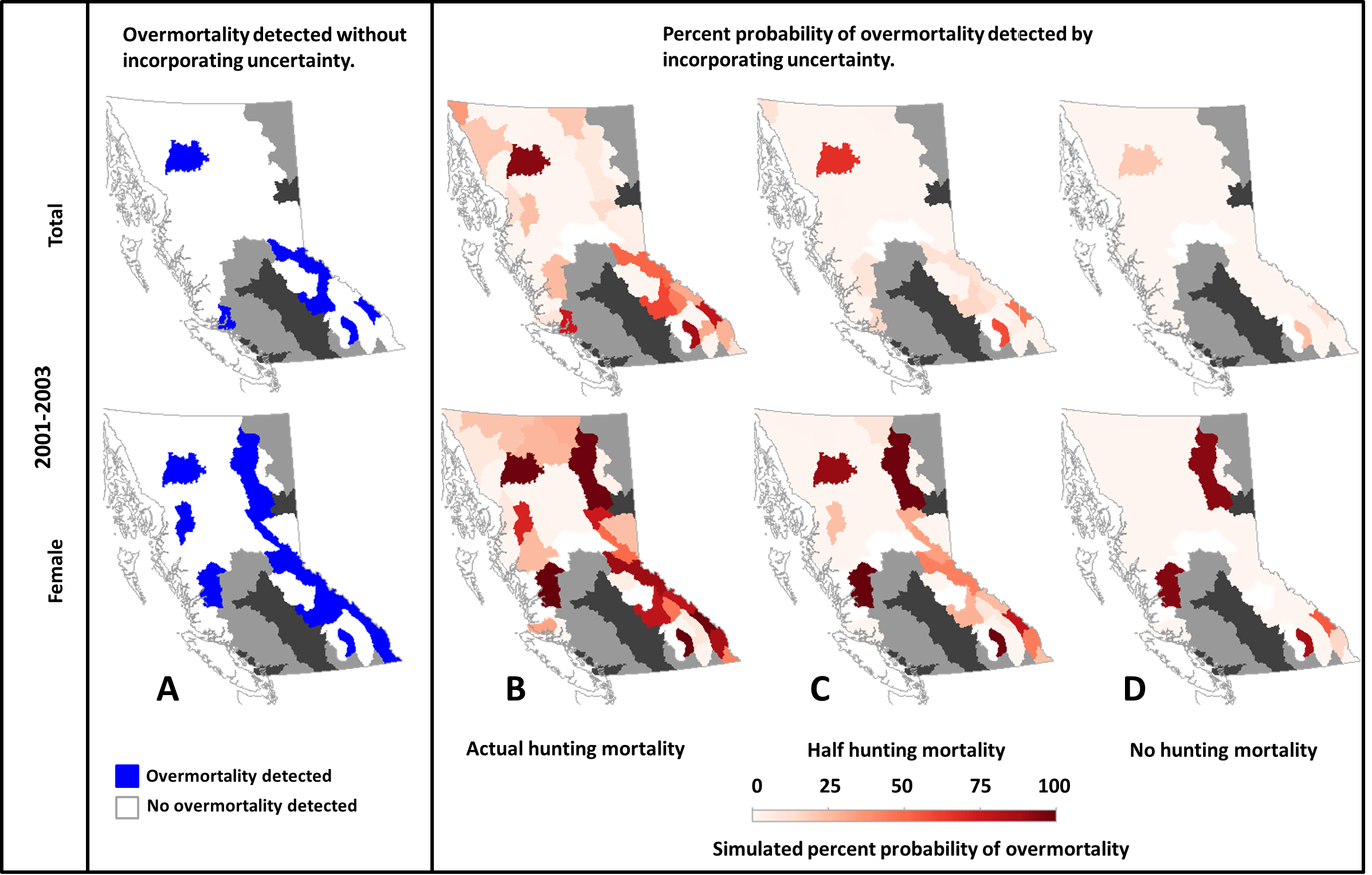

Supplement: Figure S4 — Total and female overmortalities of Grizzly Bear ( U. arctos horribilis ) Population Units (“population units”) of British Columbia, Canada, from 2001–2003. A) Overmortalities detected given known hunting mortality levels and without consideration of mortality limit uncertainty. Blue indicates population units with detected overmortality whereas white indicates population units without. B–D) Simulated probability of total or female overmortality, incorporating uncertainty around mortality limits. Panel B shows simulated probability of overmortality given known mortality rates; panels C and D show what the probability of overmortality would be had hunting mortality been reduced by 50% or 100%, respectively, assuming other sources of mortality remained unchanged. Increasingly dark red indicates an increasing probability of overmortality in a given period. Grizzly bears have been extirpated from dark-grey areas. Light-grey areas indicate population units in which populations are either threatened or were closed to hunting during the study period. (TIF) [file pone.0078041.s004.tif]

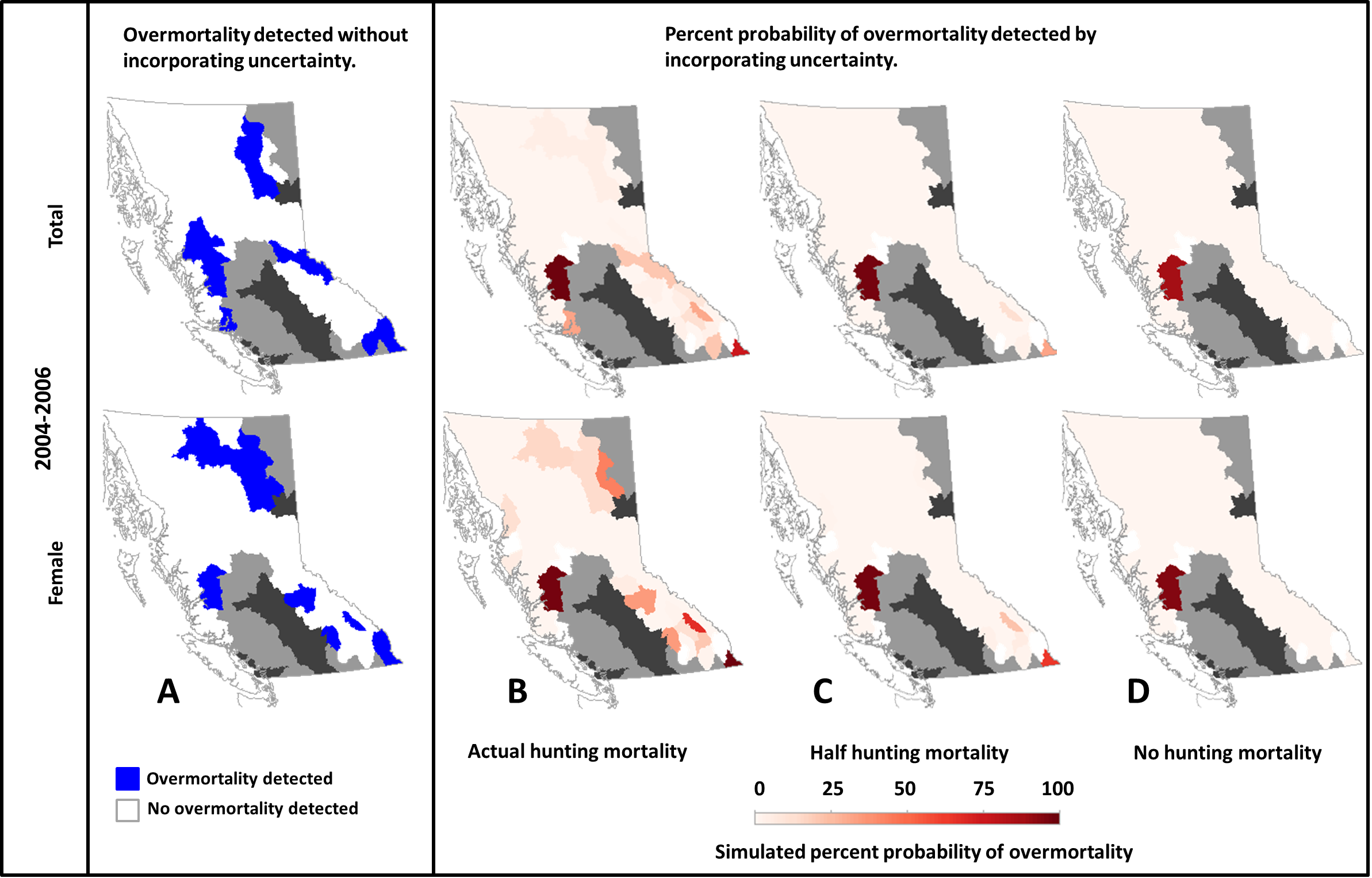

Supplement: Figure S5 — Total and female overmortalities of Grizzly Bear ( U. arctos horribilis ) Population Units (“population units”) of British Columbia, Canada, from 2004–2006. A) Overmortalities detected given known hunting mortality levels and without consideration of mortality limit uncertainty. Blue indicates population units with detected overmortality whereas white indicates population units without. B–D) Simulated probability of total or female overmortality, incorporating uncertainty around mortality limits. Panel B shows simulated probability of overmortality given known mortality rates; panels C and D show what the probability of overmortality would be had hunting mortality been reduced by 50% or 100%, respectively, assuming other sources of mortality remained unchanged. Increasingly dark red indicates an increasing probability of overmortality in a given period. Grizzly bears have been extirpated from dark-grey areas. Light-grey areas indicate population units in which populations are either threatened or were closed to hunting during the study period. (TIF) [file pone.0078041.s005.tif]
