# Supplementary material for: Confronting Uncertainty in Wildlife Management: Performance of Grizzly Bear Management
Source: PLoS One. 2013 Nov 6;8(11):e78041. doi: 10.1371/journal.pone.0078041 (PMC3819331; doi:10.1371/journal.pone.0078041)
Supplement: Appendix S1 — (DOCX) [file pone.0078041.s006.docx]

Appendix S1 - Parameters for setting mortality limits

Confronting uncertainty in wildlife management: performance of grizzly bear management

Kyle A. Artelle^1, 2^, Sean C. Anderson^1^, Andrew B. Cooper^3^, Paul C. Paquet^2, 4^, John D. Reynolds^1^, Chris T. Darimont^2, 4^

1. Earth to Ocean Research Group, Department of Biological Sciences, Simon Fraser University, Burnaby, BC, V5A 1S6, Canada

2. Raincoast Conservation Foundation, Sidney, BC, V8L 1Y2, Canada

3. School of Resource and Environmental Management, Simon Fraser University, Burnaby, BC, V5A 1S6, Canada

4. Department of Geography, University of Victoria, Victoria, BC, V8P 5C2, Canada

# Parameters for setting mortality limits

The government estimates parameters at both the Grizzly Bear Population Unit (“population unit”population unit) and Management Unit (MU) scales. We performed all analyses first at the finer MU scale, then scaled up to the population unit scale which is thought to reflect ecologically and demographically relevant sub-populations [1].

## Population estimates

The government sets population estimates using a variety of methods including: (1) DNA capture-recapture surveys [2] (approximately 14% of population units as of 2010 [3], [4]); (2) model estimates based on habitat suitability regression analyses (56% of population units as of 2010); and (3) expert opinion (30% of population units as of 2010)(see methods in Hamilton & Austin 2004b).

## Uncertainty factor

Uncertainty factors are multipliers used by the government that reduce the population estimate in some population units and periods (0% of population units in 2001-2003, 98% in 2004-2006, and 14% in 2007-2011) to account for uncertainty. These corrections, applied by provincial managers, are based on uncertainty in population estimates and acceptable levels of risk [5]. In population units inventoried with DNA, uncertainties estimated from mark-recapture were used; elsewhere, corrections were assigned according to the ratio between the current estimated density of bears and the potential habitat capability (density the habitat could support before post-contact anthropogenic landscape changes), with lower densities being assigned a higher correction factor [5]. The magnitude of corrections has been reduced in the most recent period (Appendix S2). The data used in setting factors for each individual MU or population unit are not publicly available, and if methodology has changed since 2004 it has not been publicized

## Annual allowable mortality (AAM)

Annual allowable mortality is the annual proportion of the population for which human-caused mortality is assumed sustainable by the government, informed by Harris’s [6] work in Montana that estimated maximum AAM (rate with a 10% or less probability of causing chronic declines) in highly productive populations to be somewhere between 6.35% and 6.6%. AAM values used were highest in 2007-2011 (Appendix S2).

## Period length

Period length is three years for 2001–2003 and 2004–2006, and five years for 2007–2011.

## ***Estimated unreported mortality***

Estimated unreported mortality is an estimated proportion of the population killed annually by humans through sources including poaching, illegal kills, and unreported road accidents. Sufficient data to directly assess unreported mortality exist only for the Flathead population unit. In the study period managers extrapolated unreported mortality rates to other population units by increasing or decreasing based on four factors believed to be correlated to unreported mortality: 1) human hunter density, 2) large ungulate human hunter density, 3) proportion of habitat capability in areas with roads, and 4) proportion of habitat capability in areas with human populations exceeding 5,000 within a 50 km radius [5]

## Previous period overmortalities

Previous period (total or female) overmortalities account for the number of bears killed in excess of mortality limits in the previous period. Note that the government did not carry overmortalities forward from the 2001–2003 to 2004–2006 period.

# Supporting References

1. British Columbia Ministry of Environment, Fish, Wildlife and Habitat Branch (2010) Grizzly bear hunting: frequently asked questions.

2. Hamilton AN, Austin M (2004) Estimating grizzly bear (Ursus arctos) population size in British Columbia using an expert-based approach. British Columbia Ministry of Water, Land and Air Protection. Available: http://www.env.gov.bc.ca/wld/documents/gb_est_pop_size.pdf. Accessed 25 September 2012.

3. Hamilton, A.N. (2008) 2008 Grizzly Bear Population Estimate for British Columbia.

4. Apps C (2010) The grizzly bear population inventory & monitoring strategy for British Columbia. British Columbia Ministry of Environment and Habitat Conservation Trust Foundation.

5. Austin MA, Heard DC, Hamilton AN (2004) Grizzly bear (Ursus arctos) harvest management in British Columbia. British Columbia Ministry of Water, Land and Air Protection Victoria, Canada. Available: http://www.env.gov.bc.ca/wld/documents/gb_harvest_mgmt.pdf. Accessed 25 September 2012.

6. Harris RB (1986) Modeling sustainable harvest rates for grizzly bears. Unpublished manuscript.
