# Supplementary material for: Confronting Uncertainty in Wildlife Management: Performance of Grizzly Bear Management
Source: PLoS One. 2013 Nov 6;8(11):e78041. doi: 10.1371/journal.pone.0078041 (PMC3819331; doi:10.1371/journal.pone.0078041)
Supplement: Appendix S2 — (DOCX) [file pone.0078041.s007.docx]

Appendix S2 Factors affecting detectability of overmortality

Confronting uncertainty in wildlife management: performance of grizzly bear management

Kyle A. Artelle^1, 2^, Sean C. Anderson^1^, Andrew B. Cooper^3^, Paul C. Paquet^2, 4^, John D. Reynolds^1^, Chris T. Darimont^2, 4^

1. Earth to Ocean Research Group, Department of Biological Sciences, Simon Fraser University, Burnaby, BC, V5A 1S6, Canada

2. Raincoast Conservation Foundation, Sidney, BC, V8L 1Y2, Canada

3. School of Resource and Environmental Management, Simon Fraser University, Burnaby, BC, V5A 1S6, Canada

4. Department of Geography, University of Victoria, Victoria, BC, V8P 5C2, Canada

# Appendix S2 Factors affecting detectability of overmortality

The government tests for overmortality events by comparing calculated mortality limits to recorded (*i.e.,* actual) mortality. As such, detected frequency of overmortalities can be affected by changes to recorded mortality rates or, importantly, by changes to how mortality limits are calculated (Eq 1 and Eq 2). Additionally, the temporal scale at which these comparisons are made can affect results. Specifically, longer periods might conceal overmortality events that occur over shorter periods. Accordingly, we examined two aspects that could influence detectability of overmortality events.

First, we examined the distribution of AAM estimates, unreported mortality estimates, uncertainty correction factors, and total mortality limits across allocation periods. Second, we tested the effect of allocation period length on detectability of overmortality events. We compared period overmortality to average yearly overmortality. **Period overmortality** was the number of overmortalities in a given period. **Yearly overmortality** was assessed by comparing *annual* mortality limits (period limit divided by period length) to *annual* known mortality for population unit. For each period, we also compared counts of how many population units experienced a period overmortality to counts of how many population units experienced at least one yearly overmortality.

Detection of overmortality events was likely influenced by harvest management procedures that differed among periods. We found that: i) AAM estimates increased across periods (Appendix S2 Figure 1, ii) estimates of unreported mortality decreased between 2001-2003 and subsequent periods (Appendix S2 Figure 2) and, iii) uncertainty correction factors, introduced in period 2, were considerably reduced in period 3 (Appendix S2 Figure 3). Accordingly, resultant mortality limits also increased across periods (Appendix S2 Figure 4). Whereas these changes may have reflected the inclusion of more accurate data, we note that apparent reductions in overmortality frequencies may have been due to adjusted parameters, not reduced mortality.

We also found evidence that assessing overmortality across entire periods had the effect of concealing inter-annual variation in mortality rate (Appendix S2 Figure 5); years in which mortality was unusually high were balanced by years in which it was unusually low. This concealment increased with the longer (*i.e.* five year) duration introduced in period 3, resulting in an apparent reduction of overmortality frequency compared to previous two periods, each of which were three years in length. This effect was most pronounced with total overmortality measures; whereas counts of whole period and yearly female overmortalities followed a similar pattern across periods, counts of whole period total overmortalities decreased considerably in period 3 whereas counts of yearly overmortalities did not (Appendix S2 Figure 6).


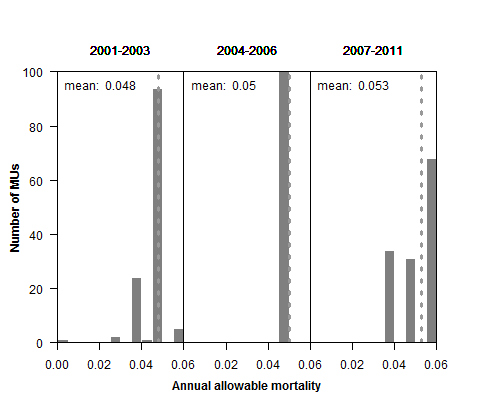


**Appendix S2 Figure 1** Management unit annual allowable mortality estimates used in grizzly bear (U. arctos horribilis) management, British Columbia, Canada, 2001-2003, 2004-2006, and 2007-2011. Dashed line represents the period mean value.


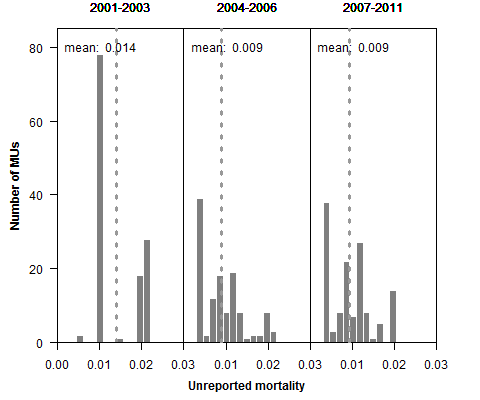


**Appendix S2 Figure 2** Management unit unreported mortality estimates used in grizzly bear *(U. arctos horribilis)* management, British Columbia, Canada, 2001-2003, 2004-2006, and 2007-2011. Dashed line represents the period mean value.


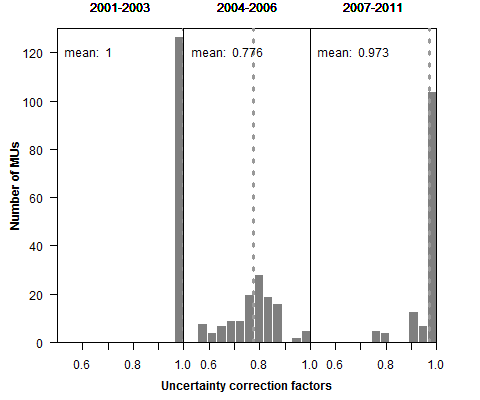


**Appendix S2 Figure 3** Management unit uncertainty correction factors used in grizzly bear *(U. arctos horribilis)* management, British Columbia, Canada, 2001-2003, 2004-2006, and 2007-2011. Dashed line represents the period mean value.


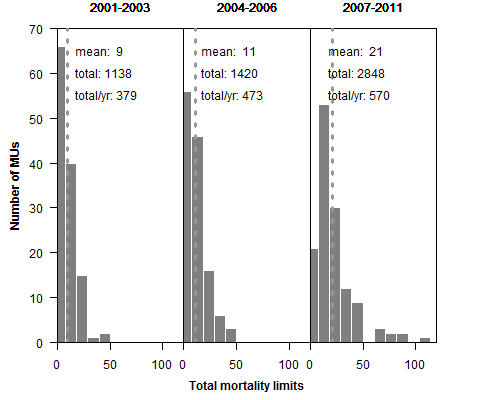


**Appendix S2 Figure 4** Management unit total mortality limit estimates used in grizzly bear *(U. arctos horribilis)* management, British Columbia, Canada, 2001-2003, 2004-2006, and 2007-2011. Dashed line represents the period mean value.


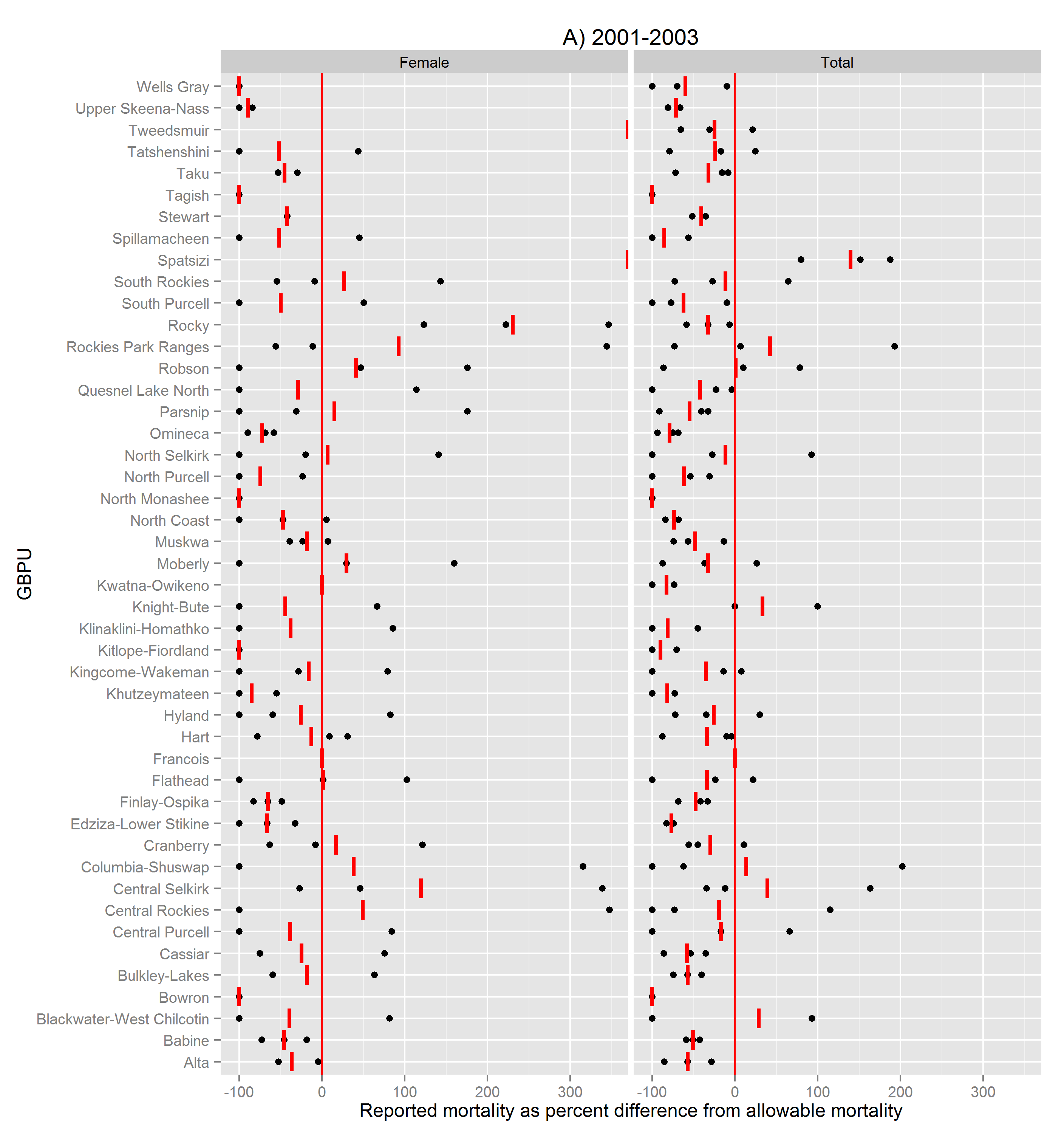


Population unit

Known mortality as percent difference from mortality target


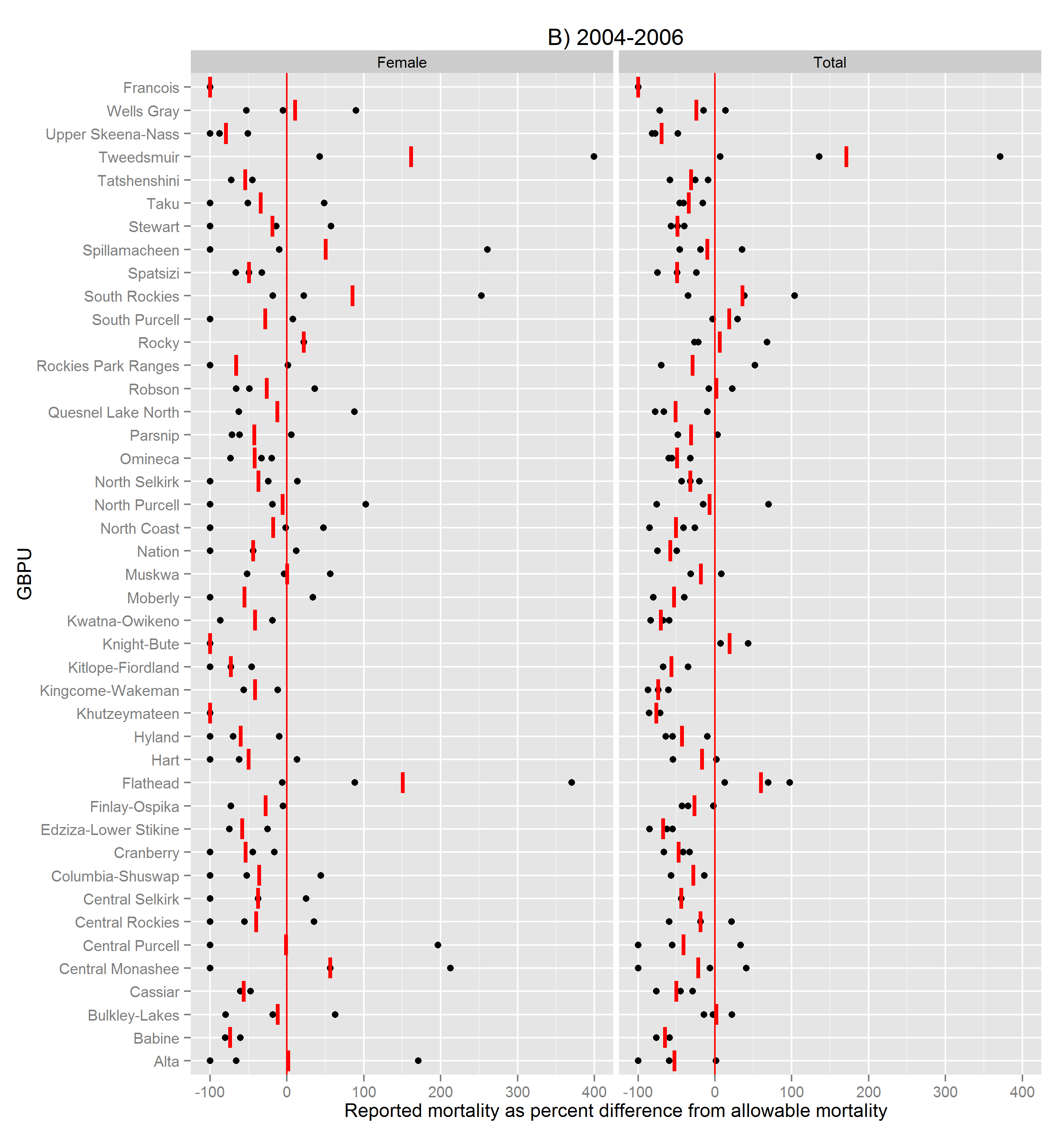


Population unit

Known mortality as percent difference from mortality target


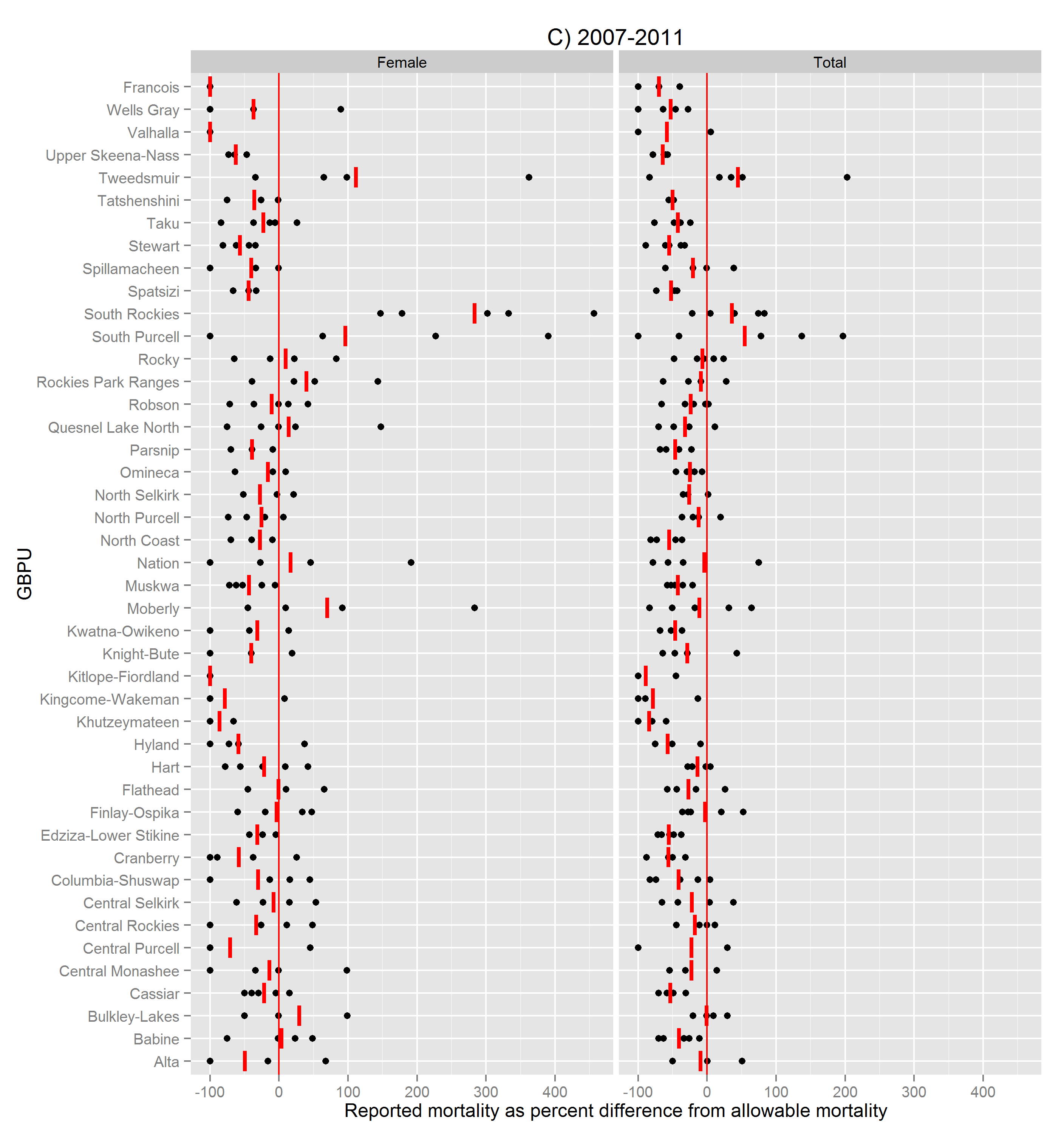


Population unit

Known mortality as percent difference from mortality target

**Appendix S2 Figure 5** Comparison of percent difference between known mortality and mortality limits in Grizzly Bear *(U. arctos horribilis)* Population Units (population units) of British Columbia, Canada, 2001-2011, at the yearly scale (black dots) and the period scale (red dashes). Vertical red line indicates a percent difference of 0 (known mortality was equal to mortality limits). Periods examined are A) 2001-2003, B) 2004-2006, and C) 2007-2011


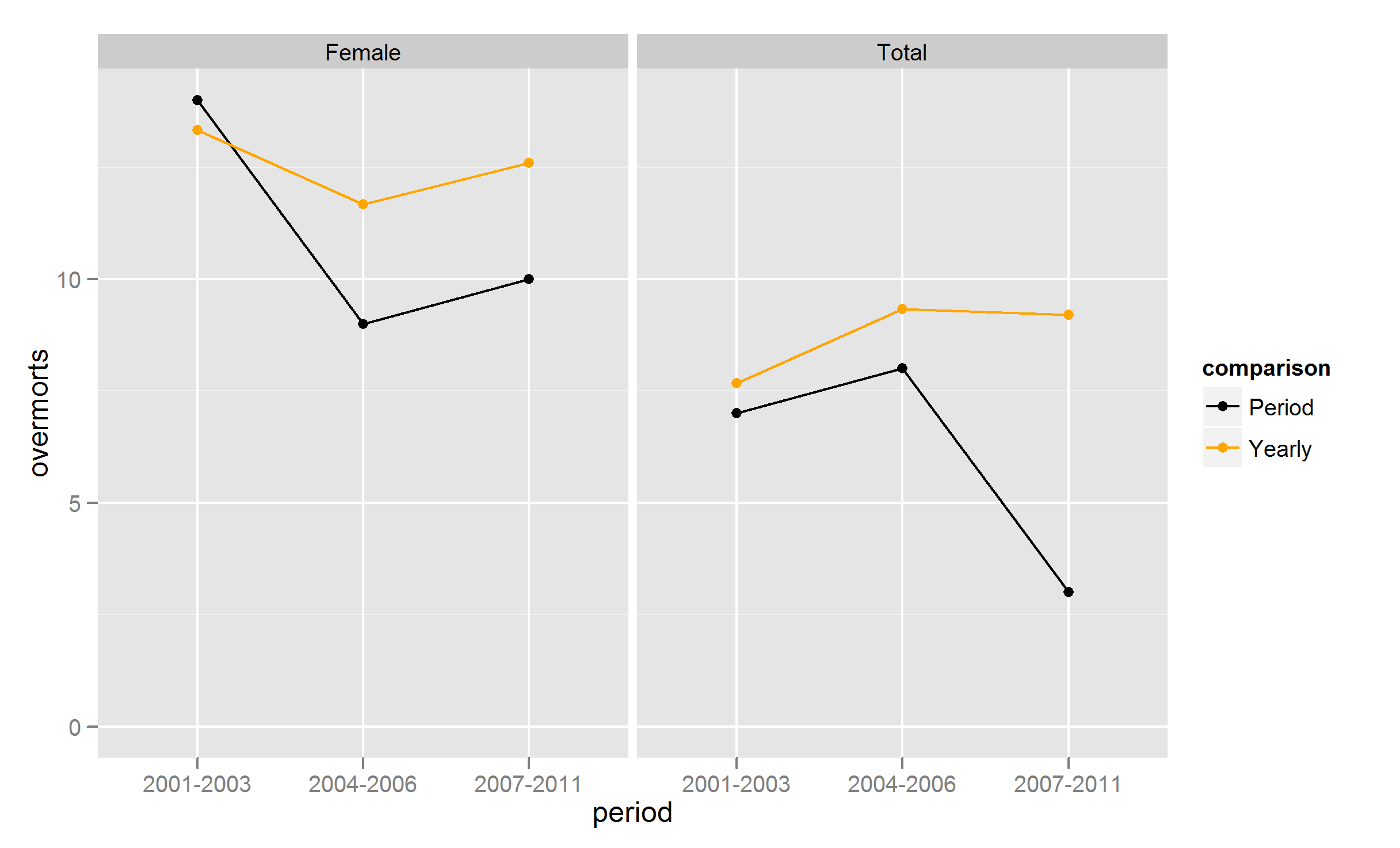


Number of population units with overmortalities

**Appendix S2 Figure 6** Comparison of detected Grizzly Bear (*U. arctos horribilis)* Population Unit (population unit) female and total overmortalities in British Columbia, Canada, from 2001-2003, 2004-2006, and 2007-2011. Black lines are number of population units with detected period overmortalities (known mortality for period exceeding mortality limits for period). Orange lines are number of population units with detected yearly overmortalities (known yearly mortality exceeded period mortality/period length at least once in given period).
