# Supplementary material for: Confronting Uncertainty in Wildlife Management: Performance of Grizzly Bear Management
Source: PLoS One. 2013 Nov 6;8(11):e78041. doi: 10.1371/journal.pone.0078041 (PMC3819331; doi:10.1371/journal.pone.0078041)
Supplement: Appendix S3 — (DOCX) [file pone.0078041.s008.docx]

Appendix S3 Parameter uncertainty ranges

Confronting uncertainty in wildlife management: performance of grizzly bear management

Kyle A. Artelle^1, 2^, Sean C. Anderson^1^, Andrew B. Cooper^3^, Paul C. Paquet^2, 4^, John D. Reynolds^1^, Chris T. Darimont^2, 4^

1. Earth to Ocean Research Group, Department of Biological Sciences, Simon Fraser University, Burnaby, BC, V5A 1S6, Canada

2. Raincoast Conservation Foundation, Sidney, BC, V8L 1Y2, Canada

3. School of Resource and Environmental Management, Simon Fraser University, Burnaby, BC, V5A 1S6, Canada

4. Department of Geography, University of Victoria, Victoria, BC, V8P 5C2, Canada

# Appendix S3 Parameter uncertainty ranges

## Population estimate

In our simulation model scenarios, we randomly increased or decreased population estimates by up to 40%. Overall, the average change in government population unit population estimates between 2004 and 2012 was approximately -10%, though adjustments ranged from -89% to 130% (Appendix S3 Figure 1A; [1], [2], and [3]). These adjusted values reflected modifications to population estimates, such as inclusion of new capture-recapture data or model refinement, not changes in the population [3]. DNA inventories (used in the estimation of approximately 14% of population units as of 2010), the most rigorous population estimates used by the government, still contain considerable uncertainty. The 95% confidence interval widths around estimates range from 33% to 152%, with an average of 70%, of the magnitude of the point estimate (Appendix S3 Figure 1B; [1], [4], and [5]). Moreover, the magnitude of uncertainty for population estimates derived from models and/or from expert opinions is untested and unknown. In summary, the ±40 % range used in our simulations is considerably narrower than that suggested by the wide confidence intervals in DNA capture-recapture estimates, large fluctuations in the government’s population unit population estimates throughout the study period, and the heavy reliance on model-derived and/or expert-based estimates.

## Annual Allowable Mortality

Annual allowable mortality (AAM) values are not empirically derived for each population unit. Instead, estimates are based on a sliding scale dictated by government estimates of a given population unit’s habitat quality and estimates of population vital rates [6], [7], with a maximum possible value of 6% (above; [8]). Importantly, this rate assumes that female mortality comprises 30% or less of the total mortality. However, published estimates of maximum AAM values vary widely. Data from Bunnel and Tait [9] modeling suggested maximum AAM for “maximally productive populations” to be 5.7%, though others considered their estimates of reproductive rates and survivorship were “very generous” [10]. Sidorowicz and Gilbert [11] estimated AAM to be 2-3% in a Yukon population, whereas simulation by McLoughlin [12] estimated maximum AAM values of 4.9% for good habitat, 2.8% for moderate, and 0% for poor habitat within BC. In the Northern Continental Divide area, Mace *et al.* [13] calculated vital rates suggesting an AAM of approximately 3%. In Yellowstone, Schwartz *et al.* [14] estimated up to 9% of female mortality could occur without population decline; however, this estimate included natural mortality, whereas the BC government AAM parameter represents only human-caused mortality. Hovey and McLellan [15] estimated maximum finite rates of increase approximately 8.5% (95% CI of 3.2-13.6%), though, similar to Schwartz *et al.* [14] this estimate is only relevant in terms of all mortality. Similarly, simulation modeling found a total mortality (human and natural) of 10 bears per year to be sustainable in an initial population of 100 bears modelled after the Yellowstone populations [16], [17], but this estimate depended on strong density-dependence for which there is mixed evidence [10]. Pease *et al.* [18] found that survival in Yellowstone grizzly populations was food-dependent, with finite rates of increase of up to 7% in years with abundant food (white-bark pine mast years), and -5% in non-mast years. Overall, population growth was likely positive, though given uncertainty in estimates the authors recommended cautious management. In our simulation model scenarios, we increased or decreased annual allowable mortality (AAM) values by up to 2% of the population estimate, resulting in maximum provincial AAMs ranging from 4% to 8%, centered on the maximum AAM point estimate of 6% currently used, a range considerably narrower than that suggested by the literature.

## Unreported Mortality

We increased or decreased unreported mortality estimates, ranging from half to twice the current point estimate for each population unit. Currently, unreported mortality estimates used by the province range from 0.3% to 2.2% of population unit populations (unpublished data). Even when explicitly studied, however, unreported mortality is notoriously difficult to estimate [19], [20]. Data from BC and beyond suggest that in many cases it could be considerably higher than estimates currently used. For example, in northwestern Alaska, less than half of grizzly bear hunting pre-1990 was thought to be reported [10], whereas wounding loss of black bears was found to account for 9% of human-caused kills on Alaska’s Kenai Peninsula from 1982-1987 [21], losses that might have occurred unknown even to the hunters. In a study of radio-collared grizzly bears in the Selkirk mountains that straddle southeastern BC, northeastern Washington, and northwestern Montana, Knick and Kasworm [22] found that the only source of death (27% of 11 bears collared) from 1983 to 1987 was illegal mortality. A meta-analysis by McLellan *et al.* [23] of grizzly mortality in the Canadian and American Rockies, 1975-1997, found that 26% of kills were illegal. These data supported the estimate reported by Peek *et al.* [19] that illegal kill of grizzlies comprised 25% of all human-caused mortality from 1970-1977. Our simulated uncertainty range, from half to twice the value of currently used estimates of unreported mortality, is therefore considerably narrower than the range suggested by the literature.

We excluded one population unit (South Rockies) from these analyses in 2004–2006 because the mortality limit for this period was set manually by the government, without record of relevant parameters. No mortalities were detected for Bowron, North Monashee, or Tagish in 2001-2003, or Francois in 2004-2006, so these were also excluded from simulated overmortality assessments.


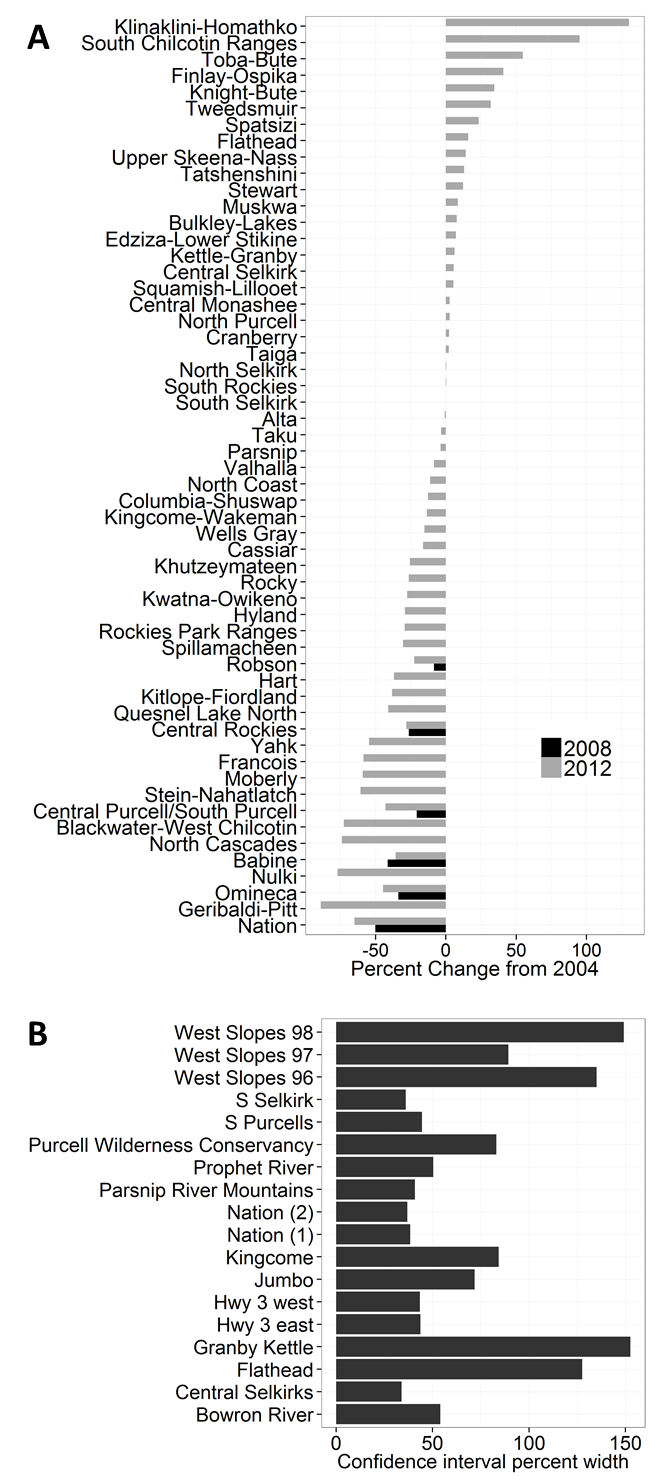


**Appendix S3 Figure 1** A) Magnitude of confidence intervals as percent of point estimate for DNA capture-recapture inventories of Grizzly Bears (*U. arctos horribilis*) in British Columbia, Canada. Data from [1], [4], and [5]. B) Percent change in estimated population size of Grizzly Bear (*Ursus arctos)* Population Units (population units) in British Columbia, Canada, from 2004 to 2008 (black bars) and 2004 to 2012 (grey bars) Data from [2], [1], and [3].

# Supporting References

1. Hamilton AN, Heard DC, Austin MA (2004) British Columbia Grizzly Bear (Ursus arctos) Population Estimate 2004.

2. Hamilton, A.N. (2008) 2008 Grizzly Bear Population Estimate for British Columbia.

3. Ministry of Forests, Lands and Natural Resource Operations (2012) British Columbia grizzly bear population estimate for 2012.

4. Proctor M, Boulanger J, Nielsen S, Servheen C, Kasworm W, et al. (2007) Abundance and density of Central Purcell, South Purcell, Yahk, and South Selkirk Grizzly Bear Population Units in southeast British Columbia. Report for BC Ministry of Environment, Nelson and Victoria, BC.

5. Mowat, G, Fear D (2004) Grizzly bear density in the Nation River area of British Columbia. Submitted to: Slocan Forest Products and B.C. Ministry of Water, Land, and Air Protection.

6. Hamilton AN, Austin MA (2002) Grizzly bear harvest management in British Columbia: background report. British Columbia Ministry of Water, Land, and Air Protection, Biodiversity Branch, Victoria, British Columbia, Canada. Available: http://www.env.gov.bc.ca/wld/documents/gbearbckgrdr.pdf. Accessed 26 September 2012.

7. Peek J, Beecham J, Garshelis D, Messier F, Miller S, et al. (2003) Management of grizzly bears in British Columbia - a review by an independent scientific panel. Submitted to: Minister of Water, Land and Air Protection Government of British Columbia Victoria, B.C.

8. Harris RB (1986) Modeling sustainable harvest rates for grizzly bears. Unpublished manuscript.

9. Bunnell FL, Tait DEN (1980) Bears in Models and in Reality: Implications to Management. Int C Bear 4: 15–23. doi:10.2307/3872837.

10. Miller S (1990) Population management of bears in North America. Int C Bear 8.

11. Sidorowicz GA, Gilbert FF (1981) The Management of Grizzly Bears in the Yukon, Canada. Wildlife Soc B 9: 125–135.

12. McLoughlin PD (2003) Managing risks of decline for hunted populations of grizzly bears given uncertainty in population parameters University of Alberta. Available: http://www.env.gov.bc.ca/wld/documents/gbear_mcl.pdf. Accessed 27 September 2012.

13. Mace RD, Carney DW, Chilton-Radandt T, Courville SA, Haroldson MA, et al. (2012) Grizzly bear population vital rates and trend in the Northern Continental Divide Ecosystem, Montana. J Wildlife Manage. Available: http://onlinelibrary.wiley.com/doi/10.1002/jwmg.250/full. Accessed 10 January 2013.

14. Schwartz CC, Haroldson MA, White GC, Harris RB, Cherry S, et al. (2006) Temporal, spatial, and environmental influences on the demographics of grizzly bears in the Greater Yellowstone Ecosystem. Wildlife Monogr 161: 1–8.

15. Hovey FW, McLellan BN (1996) Estimating population growth of grizzly bears from the Flathead River drainage using computer simulations of reproduction and survival rates. Can J Zool 74: 1409–1416. doi:10.1139/z96-156.

16. McCullough DR (1981) Population dynamics of the Yellowstone grizzly bear. In: Fowler CW, Smith TD, editors. Dynamics of large mammal populations. New York, New York: John Wiley and Sons. pp. 173–196.

17. McCullough DR (1986) The Craigheads’ data on Yellowstone grizzly bear populations: relevance to current research and management. Int C Bear: 21–32.

18. Pease CM, Mattson DJ (1999) Demography of the Yellowstone grizzly bears. Ecology 80: 957–975.

19. Peek JM, Pelton MR, Picton HD, Schoen JW, Zager P (1987) Grizzly bear conservation and management: a review. Wildlife Soc B 15: 160–169.

20. Cherry S, Haroldson MA, Robison-Cox J, Schwartz CC (2002) Estimating Total Human-Caused Mortality from Reported Mortality Using Data from Radio-Instrumented Grizzly Bears. Ursus 13: 175–184. doi:10.2307/3873198.

21. Schwartz CC, Franzmann AW (1991) Interrelationship of Black Bears to Moose and Forest Succession in the Northern Coniferous Forest. Wildlife Monogr: 3–58.

22. Knick ST, Kasworm W (1989) Shooting mortality in small populations of grizzly bears. Wildlife Soc B: 11–15.

23. McLellan BN, Hovey FW, Mace RD, Woods JG, Carney DW, et al. (1999) Rates and causes of grizzly bear mortality in the interior mountains of British Columbia, Alberta, Montana, Washington, and Idaho. The Journal of wildlife management: 911–920.
